# Prospective investigation of risk factors for prostate cancer in the UK Biobank cohort study

**DOI:** 10.1038/bjc.2017.312

**Published:** 2017-09-14

**Authors:** Aurora Perez-Cornago, Timothy J Key, Naomi E Allen, Georgina K Fensom, Kathryn E Bradbury, Richard M Martin, Ruth C Travis

**Affiliations:** 1 Cancer Epidemiology Unit, Nuffield Department of Population Health, University of Oxford, Richard Doll Building, Roosevelt Drive, Oxford OX3 7LF, UK; 2 Clinical Trial Service Unit and Epidemiological Studies Unit, Nuffield Department of Population Health, Big Data Institute, University of Oxford, Oxford OX3 7LF, UK; 3 School of Social and Community Medicine, University of Bristol, 39 Whatley Road, Bristol BS6 7QD, UK; 4 Medical Research Council/University of Bristol Integrative Epidemiology Unit, University of Bristol, Oakfield House, Oakfield Grove, Bristol BS8 2BN, UK; 5 National Institute for Health Research Bristol Biomedical Research Unit in Nutrition, Bristol Education & Research Centre, Upper Maudlin Street, Bristol BS2 8AE, UK

**Keywords:** risk factors, prostate cancer, cohort study, prospective, UK Biobank

## Abstract

**Background:**

Prostate cancer is the most common cancer in British men but its aetiology is not well understood. We aimed to identify risk factors for prostate cancer in British males.

**Methods:**

We studied 219 335 men from the UK Biobank study who were free from cancer at baseline. Exposure data were collected at recruitment. Prostate cancer risk by the different exposures was estimated using multivariable-adjusted Cox proportional hazards models.

**Results:**

In all, 4575 incident cases of prostate cancer occurred during 5.6 years of follow-up. Prostate cancer risk was positively associated with the following: black ethnicity (hazard ratio black *vs* white=2.61, 95% confidence interval=2.10–3.24); having ever had a prostate-specific antigen test (1.31, 1.23–1.40); being diagnosed with an enlarged prostate (1.54, 1.38–1.71); and having a family history of prostate cancer (1.94, 1.77–2.13). Conversely, Asian ethnicity (Asian *vs* white hazard ratio=0.62, 0.47–0.83), excess adiposity (body mass index (⩾35 *vs* <25 kg m^−2^=0.75, 0.64–0.88) and body fat (⩾30.1 *vs* <20.5%=0.81, 0.73–0.89)), cigarette smoking (current *vs* never smokers=0.85, 0.77–0.95), having diabetes (0.70, 0.62–0.80), and never having had children (0.89, 0.81–0.97) or sexual intercourse (0.53, 0.33–0.84) were related to a lower risk.

**Conclusions:**

In this new large British prospective study, we identified associations with already-established, putative and possible novel risk factors for being diagnosed with prostate cancer. Future research will examine associations by tumour characteristics.

Prostate cancer is the most common cancer in men in the United Kingdom (UK), with 40 300 cases diagnosed in 2015 (ONS, 2017). The aetiology of prostate cancer is not well understood, and the well-established risk factors age, ethnicity, and family history of the disease are not modifiable (Cuzick *et al*, 2014; WCRF/AICR, 2014). However, there is evidence that circulating insulin-like growth factor-I (IGF-I), which is influenced by environmental factors, is related to higher prostate cancer risk (Travis *et al*, 2016), and the latest World Cancer Research Fund meta-analysis of prospective studies concluded that it is probable that obesity is associated with a higher incidence of aggressive prostate cancer. While there is limited epidemiological evidence for other lifestyle risk factors for prostate cancer (WCRF/AICR, 2014), the relatively high variation in incidence rates worldwide suggests that differences in exposure to environmental factors may have a role in prostate cancer development, although some of the variation is due to differences between countries in prostate-specific antigen (PSA) testing (Ferlay *et al*, 2015).

The UK Biobank cohort is an important new resource for the study of cancer aetiology. We report here the first results from this cohort on the association between prostate cancer incidence and potential risk factors, including socio-demographic, anthropometric and lifestyle factors, health status, prostate-specific factors prior to the recruitment, sexual history, early life characteristics, hair colour, and balding pattern. We also tested whether these associations vary by time to diagnosis.

## Materials and methods

### Study design

The UK Biobank is a prospective study designed to be a resource for research into the causes of disease in middle and old age. The study protocol and information about data access are available online (http://www.ukbiobank.ac.uk/wp-content/uploads/2011/11/UK-Biobank-Protocol.pdf) and more details of the recruitment and study design have been published elsewhere (Sudlow *et al*, 2015). In brief, all participants were registered with the UK National Health Service (NHS) and lived within ∼25 miles (40 km) of one of the assessment centres. The UK Biobank invited ∼9.2 million people to participate through postal invitation with a telephone follow-up, with a response rate of 5.7%. A total of 503 317 men and women aged 40–69 years were recruited in 22 assessment centres across England, Wales and Scotland, between 2006 and 2010. In all, 608 participants have subsequently withdrawn from the study and their data were not available for analysis. The UK Biobank study was approved by the North West Multi-Centre Research Ethics Committee (reference number 06/MRE08/65), and at recruitment all participants gave informed consent to participate in UK Biobank and be followed-up, using a signature capture device.

After excluding 9835 men with prevalent cancer (except C44: non-melanoma skin cancer), and 2 men censored on entry day, these analyses included a total of 219 335 men (Supplementary Figure 1).

### Exposure assessment

Participants provided detailed self-reported data via a touch screen questionnaire and a verbal interview with a trained nurse at the assessment centres at baseline (Sudlow *et al*, 2015), and a wide range of physical measurements (e.g., body mass index (BMI) and including bioimpedance) and biological samples were collected (Sudlow *et al*, 2015). Information about the assessment procedure is available at http://www.ukbiobank.ac.uk/.

Exposure data included information on socio-demographic factors (region, Townsend deprivation index, education level, ethnicity, employment, and living with a wife or partner), anthropometric measurements (standing height, weight, BMI, percentage body fat, waist and hip circumferences, waist to hip ratio (WHR) (UK-Biobank, 2014)), lifestyle characteristics (smoking status, alcohol consumption, and physical activity), health-related factors (vasectomy, hypertension, and diabetes), prostate-specific factors prior to recruitment (PSA test, enlarged prostate, and family history of prostate cancer), sexual history (number of children, age at first sexual intercourse, lifetime heterosexual partners, same-sex intercourse, and lifetime number of same-sex partners), early life factors (puberty as defined by age of first facial hair, relative age voice broke, and comparative body size and height at age 10 years), and hair colour and balding pattern. Detailed information regarding how these variables were collected is given in the Supplementary Methods.

### Outcome assessment

Men were followed-up until the censoring date (30 September 2014 in England and Wales, and 31 December 2014 in Scotland) via record linkage to the NHS Central Register, which provides information on cancer registrations and deaths. The end point included in these analyses is first diagnosis of prostate cancer (International Classification of Diseases Tenth revision codes: C61; (WHO, 2010)) or death from prostate cancer, whichever was first. Person-years were calculated from the date of recruitment to the date of cancer registration (first malignant neoplasm, except non-melanoma skin cancer (ICD-10 C44)), death, or the censoring date, whichever occurred first.

### Statistical analysis

Cox proportional hazards models were used to calculate hazard ratios (HRs) and 95% confidence intervals (CIs) for prostate cancer risk, using age as the underlying time variable. All analyses were stratified by geographical region of recruitment (10 UK regions, except for when region was the main exposure of interest) and age (<45, 45–49, 50–54, 55–59, 60–64, ⩾65 years) at recruitment. Exposure variables (socio-demographic factors, anthropometric measurements, lifestyle characteristics, health status, prostate-specific factors prior to recruitment, sexual history, early life factors, and hair colour and balding pattern) were divided into categories based on their distribution at baseline in the whole cohort (categories for each exposure are explained in detailed in Supplementary Methods). Missing and/or unknown values of the exposure of interest were not included in the analyses, but missing and/or unknown values were assigned to a separate category when the variable was included as a covariate. As these are the first analyses on the association between potential risk factors and incidence of total prostate cancer in UK Biobank, potential confounders were first identified *a priori* based on possible risk factors for prostate cancer that have some support in the literature (Sutcliffe & Colditz, 2013; Cuzick *et al*, 2014; WCRF/AICR, 2014; Rider *et al*, 2016).

Minimally adjusted Cox regression models were performed to identify statistically important covariates from the *a priori* potential confounders. On the basis of results from the minimally adjusted Cox regression analyses, the multivariable-adjusted model was additionally adjusted for Townsend deprivation score (fifths, unknown (0.1%)), ethnicity (white, mixed background, Asian, black, other, and unknown (0.7%)), lives with a wife or partner (no, yes), BMI (<25, ⩾25–<30, ⩾30–<35, ⩾35 kg m^−2^, and unknown (0.6%)), cigarette smoking (never, former, current, and unknown (0.7%)), physical activity (low (0–<10 metabolic equivalents (METs) per week), moderate (⩾10–<50 METs per week), high (⩾50 METs per week), and unknown (3.7%)), diabetes (no, yes, and unknown (0.6%)), enlarged prostate (no or unknown, and yes), and family history of prostate cancer (no, yes (brother or father), and unknown (45.1%)). For each adjustment variable, missing values were assigned to a separate category. Body mass index was not included in the multivariable-adjusted model when fat mass, waist circumference, and WHR were the main exposure variables.

*P*-values from the multivariable-adjusted model for the association of each exposure with prostate cancer risk were calculated as follows: *P*-values for likelihood ratio tests for variables with more than two categories (categorical variables) were obtained comparing the model with and without the variable of interest; *P*-values for dichotomous variables were obtained comparing the reference category to the other category in the model; and *P*-values for trend were obtained using a pseudo-continuous variable equal to the median value in each category for continuous variables. The proportional hazards assumption was tested using time-varying covariates and Schoenfeld residuals and revealed no evidence of deviation from the proportional hazards assumption.

Sensitivity analyses were performed to test for heterogeneity in the associations with risk by time between recruitment and diagnosis (<2 and ⩾2 years) to examine whether there were associations for cancers diagnosed shortly after recruitment, which could indicate reverse causality. For this purpose, we fitted stratified Cox models based on competing risks and compared the risk coefficients and s.e.’s in the subgroups of interest (<2 or ⩾2 years between recruitment and diagnosis). All analyses were performed using Stata version 14.1 (Stata Corporation, College Station, TX, USA), all tests of significance were two-sided, and *P*-values <0.05 were considered statistically significant.

## Results

### Participants’ characteristics

A total of 4575 men were diagnosed with prostate cancer after a mean 5.6 years (s.d., 1.0 years) follow-up. Table 1 shows the characteristics of the study population at baseline. The mean age at recruitment was 56.5 years (s.d., 8.2 years) and the mean BMI was 27.8 kg m^−2^ (s.d., 4.2). Among all participants, 12.4% reported that they were current cigarette smokers, and 43.2% reported drinking at least 20 g of alcohol per day. Physical inactivity was reported by 27.6% of men. Diabetes was reported by 6.9% of men. Regarding prostate-specific factors prior to recruitment, 27.6% of men reported having had at least one PSA test and 7.5% of men had a family history of prostate cancer.

Table 2 shows the HRs of prostate cancer in relation to socio-demographic factors, anthropometric factors, and lifestyle factors before and after adjusting for multiple factors. There were no marked differences between the minimally and multivariable-adjusted models. After multivariable adjustment, men living in North-West England, North-East England, Yorkshire & the Humber, and in South-West England were significantly less likely to be diagnosed with prostate cancer than men living in London. There was no evidence for an association between living in a deprived area, having higher education, being unemployed, or living with a partner and prostate cancer risk. Compared to men of white ethnicity, Asians had a lower risk (HR=0.62, 95% CI 0.47–0.83) and black men had a higher risk (2.61, 2.10–3.24) of prostate cancer.

Height was not associated with prostate cancer risk in the multivariable-adjusted model (Table 2). Obesity (BMI ⩾30–<35 *vs* <25 kg m^−2^: HR=0.88, 95% CI 0.81–0.97) and morbid obesity (BMI ⩾35 *vs* <25 kg m^−2^=0.75, 0.64–0.88), high body fat percentage (HR for the highest *vs* the lowest fifth=0.81, 0.73–0.89), high waist circumference (HR for the highest *vs* the lowest fifth=0.90, 0.82–0.99), and high WHR (HR for the highest *vs* the lowest fifth=0.87, 0.79–0.96) were all significantly inversely associated with prostate cancer risk.

Among the lifestyle characteristics analysed in this study, compared with never smokers, current cigarette smokers (0.85, 0.77–0.95) and former cigarette smokers (0.93, 0.88–0.99) had a significantly lower risk of prostate cancer, while no association with risk was observed for alcohol intake or physical activity.

Table 3 shows the HRs of prostate cancer in relation to health status and prostate-specific factors prior to recruitment, sexual history, early life factors, and hair colour and balding pattern. Men with a self-reported diagnosis of diabetes had a lower risk of incident prostate cancer (HR=0.70, 0.62–0.80). In contrast, men who had had a PSA test prior to recruitment (1.31, 1.23–1.40), had any first-degree family history of prostate cancer (1.94, 1.77–2.13), and who reported that they had been diagnosed with an enlarged prostate (1.54, 1.38–1.71) had an elevated risk of prostate cancer. Moreover, compared to men with no family history of prostate cancer, men with both their father and brother diagnosed with prostate cancer had an even higher risk of prostate cancer (3.35, 2.33–4.81).

For sexual history factors, men who had no children had a reduced prostate cancer risk (never *vs* ever, HR=0.89, 95% CI 0.81–0.97), as did men who reported they had never had sex (never *vs* ever, 0.53, 0.33–0.84). Other sexual history characteristics were not related to prostate cancer risk (Table 3).

Early life factors (relative age of first facial hair, relative age voice broke, and comparative body size and height at age 10) and hair colour and pattern were not associated with prostate cancer risk (Table 3).

For most factors, there was no significant heterogeneity in the association of prostate cancer risk according to time between recruitment and diagnosis (<2 and ⩾2 years) (Supplementary Table 1). However, there was evidence of heterogeneity by time to diagnosis for the association of prostate cancer risk with ethnicity (*P*_heterogeneity_=0.001; for black *vs* white, HR=3.94, 2.85–5.44 for men diagnosed within 2 years and HR=2.01, 1.49–2.70 for those diagnosed after 2 years), unemployment (*P*_heterogeneity_<0.001; HR=1.34, 1.18–1.51 in the first 2 years and HR=0.89, 0.82–0.97 after 2 years), hypertension (*P*_heterogeneity_=0.018; for yes *vs* no, HR=1.12, 1.00–1.24 in the first 2 years and HR=0.95, 0.89–1.02 after 2 years), having had a PSA test prior to recruitment (*P*_heterogeneity_=0.003; HR=1.51, 1.35–1.70 in the first 2 years and 1.23, 1.14–1.33 after 2 years), and having had enlarged prostate (*P*_heterogeneity_<0.001; HR=2.16, 1.82–2.56 in the first 2 years and 1.27, 1.10–1.46 after 2 years; Supplementary Table 1).

## Discussion

Here we report results on associations with established and putative risk factors for prostate cancer risk in a large prospective study of British men. We found that black ethnicity and having previously had a PSA test, an enlarged prostate, or a family history of prostate cancer were positively associated with prostate cancer risk. The risk of being diagnosed with prostate cancer was lower in those who were of Asian ethnic origin, and in men who had obesity, smoked cigarettes, had diabetes, and had never had sex. Time to diagnosis was not a strong modifier of these associations, although men who had had a PSA test prior to recruitment were more likely to be diagnosed with prostate cancer in the first 2 years of follow-up.

### Ethnicity, socio-demographic, anthropometric and lifestyle factors, and risk of prostate cancer

Our findings for ethnicity accord with findings from the retrospective PROCESS cohort study, which has previously shown that compared to white men, black men in southern England have a greater risk of being diagnosed with prostate cancer (Ben-Shlomo *et al*, 2008), while Asian men have a lower risk (Metcalfe *et al*, 2008). A cross-sectional study within the Hospital Episodes Statistics database for England also showed a higher risk of prostate cancer in men with black ethnicity (Maruthappu *et al*, 2015).

Geographic differences in prostate cancer incidence rates have been observed in the United States (Cook *et al*, 2015), indicating that risk factors for prostate cancer occurrence and for diagnosis may vary geographically. In the current British study, there were differences in risk between certain geographical regions. It is possible that some of the regional differences might be due to differences in detection rates of asymptomatic prostate cancer (Littlejohns *et al*, 2016). Socioeconomic status, education level, employment status, and marital status (living with a wife or partner) were not associated with prostate cancer risk.

While we did not observe a significant association of prostate cancer risk with height, as seen in previous prospective studies (Pischon *et al*, 2008; WCRF/AICR, 2014), our results did show that men with higher BMI and fat mass percentage had a lower risk of prostate cancer. While two previous prospective studies have used fat mass measurement from bioimpedance measurements (477 (MacInnis *et al*, 2003) and 817 incident cases (Wallstrom *et al*, 2009), respectively), this is to our knowledge the first large prospective study using bioimpedance to estimate body composition. Previous prospective investigations have also found a link between excess adiposity, typically as estimated by BMI or waist circumference, and a lower risk of overall prostate cancer risk (Perez-Cornago *et al*, 2017; WCRF/AICR, 2014). It is possible that this association might be due to detection bias as in this cohort men with obesity are less likely to have had a PSA test (Littlejohns *et al*, 2016). Moreover, previous studies have reported slightly lower PSA concentrations in men with high BMI (Bonn *et al*, 2016). A positive association between adiposity and risk for aggressive prostate cancer has also been observed in previous studies (WCRF/AICR, 2014), but data on stage and grade are not yet available in the UK Biobank cohort.

Findings from the present study of a nearly 15% reduced risk of prostate cancer in cigarette smokers compared to never smokers are consistent with results from a 2010 meta-analysis of 24 observational studies (Huncharek *et al*, 2010). However, men who were current smokers were markedly less likely to have had a PSA test than never smokers in UK Biobank (Littlejohns *et al*, 2016), and this association might therefore be due to detection bias.

In agreement with findings from a recent meta-analysis (WCRF/AICR, 2014), alcohol consumption was not related to prostate cancer risk in the current study. Total physical activity was also not associated with prostate cancer risk in the current study, whereas findings from a recent meta-analysis of 46 890 incident cases, which showed that greater leisure-time physical activity was associated with a higher risk of prostate cancer (Moore *et al*, 2016), although the impact on those results of detection bias was not clear (i.e., the extent to which PSA testing is associated with health-conscious behaviour).

### Health status, prostate-specific factors prior to recruitment, sexual history, early life factors, and hair colour and pattern

In agreement with previous studies (Byrne *et al*, 2017; Nayan *et al*, 2016), we found no association between vasectomy status and prostate cancer risk. In the current study, hypertension was not linked to prostate cancer risk, although there was some evidence that it was associated with an increased risk in the first 2 years of follow-up. A recent meta-analysis has suggested that hypertension may be related to prostate cancer incidence, but high heterogeneity among studies was noted (Liang *et al*, 2016), and more prospective data are needed. Our finding that diabetes was associated with a reduced risk of prostate cancer has been consistently reported in other cohort studies (Rodriguez *et al*, 2005; Tsilidis *et al*, 2015). It has been suggested that the inverse association between diabetes and prostate cancer risk might be due to lower circulating concentrations of IGF-I (Teppala & Shankar, 2010) and/or testosterone (Grossmann, 2011) or to potential anti-carcinogenic properties of diabetes medication (Wright and Stanford, 2009). Information was not available on diabetes type for the current analyses, but the majority of cases in this age group will be of type II diabetes (Kirkman *et al*, 2012).

Increases in the proportion of men undergoing PSA testing in the UK have led to a large increase in prostate cancer diagnoses over recent decades (Lilja *et al*, 2008), and as expected, history of having had a PSA test was positively associated with prostate cancer risk in our study. As expected, previous PSA testing was also more strongly associated with risk in the first 2 years of follow-up, owing to it being a first-line test in the diagnostic pathway for men with prostatic symptoms. Similarly, men with an enlarged prostate were also more likely to be diagnosed with prostate cancer, particularly within the first 2 years, suggesting increased likelihood of cancer detection following urological investigations.

This study found that having a first-degree relative with prostate cancer doubled the risk of prostate cancer, which is well-established, although to date only approximately one-third of this risk is explained by known genetic variants (Benafif & Eeles, 2016). The risk was even higher in men where both the father and the brother had prostate cancer, although the number of cases in this category was small (*n*=30). The remainder of the excess familial risk may be due to a combination of currently unidentified genetic variation, shared environmental factors, and differential detection in family members (e.g., health-seeking behaviours).

There are few prospective data on sexual history and prostate cancer risk (Rosenblatt *et al*, 2001). Our results show that men who reported never having had sex have a lower prostate cancer risk than men who had ever had sex. Moreover, men who had not had children had a lower risk compared to those who had had children. Men who have not had sex might have erectile dysfunction or low sexual interest, which are both conditions that have been linked to reduced circulating androgen levels (Isidori *et al*, 2005). Observational epidemiological studies to date have not shown an association between circulating testosterone and prostate cancer risk (Roddam *et al*, 2008), but more data are required to examine risk in men with very low circulating testosterone levels.

To date it is unclear whether early-life exposures are involved in prostate cancer aetiology (Sutcliffe & Colditz, 2013; Moller *et al*, 2015; Sarre *et al*, 2016), although it has been speculated that childhood body size or timing of puberty may be related to changes in prostate tissue in early adulthood (Sutcliffe & Colditz, 2013; Sarre *et al*, 2016). Mendelian randomisation studies have shown an association between genetically determined age at puberty and higher risk of aggressive prostate cancer risk (Bonilla *et al*, 2016). In the current study, however, we found no evidence for an association between a number of early life factors (relative age of first facial hair, relative age voice broke, and comparative height and body size at age 10) and the future risk of prostate cancer.

It has been hypothesised that pigmentation-related traits may influence prostate cancer risk, possibly through altered vitamin D synthesis, owing to a finding in the Alpha-Tocopherol, Beta-Carotene Cancer Prevention Study that men with naturally red hair (which is determined by polymorphisms in the melanocortin-1-receptor (*MC1R*) gene) had a lower risk of prostate cancer compared to men with light brown hair (Weinstein *et al*, 2013); however, no significant association between naturally red hair and prostate cancer risk was observed in the current study. Because of the influence of the active androgen dihydrotestosterone on both the growth of prostate cells and on androgenic alopecia, male-pattern balding has also been suggested as possible risk factor for prostate cancer, although the findings are inconclusive (Muller *et al*, 2013; Zhou *et al*, 2015a, 2015b; Sarre *et al*, 2016) and the current study found no association between balding pattern and prostate cancer risk.

### Study strengths and limitations

This is, to our knowledge, the largest single prospective study of risk factors for prostate cancer in British men. The UK Biobank has collected detailed information on numerous possible prostate cancer risk factors, including risk factors that few previous studies have examined in detail, such as body fat mass, sexual history, early life factors, and hair colour and pattern. In particular, fat mass estimated using bioimpedance is a better marker of overall adiposity than BMI or waist circumference, which does not differentiate between muscle and fat mass. Moreover, many risk factors, such as adiposity measurements or blood pressure, were assessed by trained research clinic staff instead of being self-reported.

Despite the breadth of the exposure information collected at recruitment, we cannot exclude the possibility of residual confounding by unknown or unmeasured factors. In addition, because of the number of tests performed, some of the associations observed might be due to chance. For some of the rare exposures (e.g., red hair colour or never having had sexual intercourse), there are small numbers of exposed cases for robust analysis. This cohort includes participants from multiple regions, including deprived areas, but it is not a representative sample of the whole UK population (Fry *et al*, 2017). However, it does include participants with a wide range of exposures for a comprehensive set of characteristics allowing internally valid and informative comparisons of risk by factors of interest. Although the number of missing values in our cohort is low (<1%), there are some variables, such as family history of prostate cancer, that have a higher proportion of missing values. These values may not be missing at random, for example, participants with family history of prostate cancer may have replied to this question, while participants with no family history may have left this question blank. Finally, risk factors for prostate cancer may differ by tumour characteristics, but data on tumour stage and Gleason grade were not available for the current analysis. We will perform analyses by subgroups of disease aggressiveness when these data become available.

We have reported a range of established and novel risk factors that are associated with subsequent prostate cancer risk in this large UK prospective study. In particular, black ethnicity, having had a PSA test, an enlarged prostate, and a family history of prostate cancer were positively associated with prostate cancer risk, while Asian ethnicity, obesity, smoking status, diabetes, and never having had children or sexual intercourse were related to a lower prostate cancer risk. Future research in UK Biobank will include analyses by disease aggressiveness to explore whether these associations are due to differences in the likelihood of being diagnosed and/or differences in the risk of developing clinically important prostate cancer.

## Figures and Tables

**Table 1:** Baseline characteristics of all men included in the analysis and of men who developed prostate cancer

**Characteristics**	**All men**	**Men who developed prostate cancer**
No. of men	219 335	4575
Socio-demographic		
Age at recruitment (years), mean (s.d.)	56.5 (8.2)	62.4 (5.2)
Most deprived quintile, % (*n*)	20.0 (43 809)	15.7 (719)
No qualifications, % (*n*)	13.5 (29 593)	11.5 (528)
Black ethnicity, % (*n*)	1.5 (3279)	1.9 (89)
Not in paid/self-employment, % (*n*)	38.9 (85 223)	41.6 (1904)
Living with partner, % (*n*)	76.1 (166 967)	79.3 (3630)
Anthropometric		
Height (cm), mean (s.d.)	175.6 (6.8)	175.1 (6.7)
BMI (kg m^−2^), mean (s.d.)	27.8 (4.3)	27.5 (3.8)
Body fat (%), mean (s.d.)	25.3 (5.8)	25.4 (5.5)
Waist circumference (cm), mean (s.d.)	96.9 (11.4)	96.9 (10.6)
Waist to hip ratio, mean (s.d.)	0.936 (0.065)	0.939 (0.064)
Lifestyle		
Current cigarette smokers, % (*n*)	12.4 (27 428)	9.0 (417)
Drinking alcohol ⩾20 g per day, % (*n*)	43.2 (94 750)	42.2 (1930)
Low physical activity (0–<10 METs per week), % (*n*)	27.6 (60 589)	26.1 (1196)
Self-reported health status		
Vasectomy, % (*n*)	5.2 (11 366)	5.1 (232)
Hypertension, % (*n*)	52.1 (114 193)	59.0 (2700)
Diabetes, % (*n*)	6.9 (15 191)	5.9 (270)
Prostate specific factors prior to recruitment		
Ever had a PSA test, % (*n*)	27.6 (60 646)	46.6 (2133)
Enlarged prostate, % (*n*)	3.2 (7093)	7.8 (359)
Family history of prostate cancer, % (*n*)	7.5 (16 449)	13.1 (599)
Sexual history		
Number of children, median (s.d.)	2.0 (1.4)	2.0 (1.3)
Age at first sexual intercourse (years), median (s.d.)	18.0 (6.2)	19.0 (6.2)
Lifetime number of heterosexual partners, median (s.d.)	3.0 (92.2)	2.0 (45.1)
Same-sex intercourse, % (*n*)	3.9 (8576)	3.2 (146)
Lifetime number of same-sex partners, median (s.d.)	2.0 (384.9)	2.0 (196.4)
Early life		
Age at first facial hair older than average, % (*n*)	12.3 (27 009)	10.0 (459)
Age voice broke older than average, % (*n*)	5.4 (11 861)	4.2 (194)
Plumper than average at age 10, % (*n*)	13.4 (29 305)	11.7 (536)
Taller than average at age 10, % (*n*)	24.6 (53 953)	24.4 (1115)
Hair colour and pattern		
Red hair colour, % (*n*)	3.5 (7726)	3.1 (144)
Hair/balding pattern 4,^a^ % (*n*)	17.8 (39 051)	19.3 (882)

Abbreviations: BMI=body mass index; PSA=prostate-specific antigen.

Percentages do not sum to 100% due to missing data.

a Pattern 4, complete balding.

**Table 2:** Hazard ratios (95% CI) for prostate cancer in relation to various socio-demographic, anthropometric, and lifestyle factors at baseline

**Characteristics**	**Total** ***n***	**Cases**	**HR (95% CI) minimally adjusted**^a^	**HR (95% CI) multivariable-adjusted**^b^	***P*****-value**^c^
**Socio-demographic**					
Region					0.001
London	29 357	549	1 ref	1 ref	
Wales	9075	201	0.87 (0.74–1.02)	0.88 (0.75–1.04)	
North-West England	35 235	749	0.84 (0.76–0.94)	0.86 (0.76–0.96)	
North-Eastern England	25 519	480	0.77 (0.68–0.87)	0.78 (0.69–0.89)	
Yorkshire & the Humber	32 574	635	0.87 (0.77–0.97)	0.86 (0.77–0.97)	
West Midlands	20 957	459	0.99 (0.87–1.12)	0.99 (0.87–1.12)	
East Midlands	14 792	326	0.90 (0.78–1.03)	0.89 (0.77–1.02)	
South-East England	18 423	471	0.98 (0.87–1.11)	0.95 (0.84–1.08)	
South-West England	18 181	332	0.83 (0.72–0.95)	0.80 (0.70–0.92)	
Scotland	15 222	373	0.86 (0.75–0.98)	0.87 (0.76–1.00)	
Townsend deprivation score, fifths					0.12
One (most affluent)	43 855	1020	1 ref	1 ref	
Two	43 765	1018	1.02 (0.94–1.11)	1.03 (0.94–1.12)	
Three	43 808	971	1.00 (0.92–1.10)	1.03 (0.94–1.12)	
Four	43 808	842	0.94 (0.86–1.03)	0.98 (0.89–1.07)	
Five (most deprived)	43 809	719	0.86 (0.78–0.95)	0.91 (0.82–1.01)	
Education					0.665
No qualifications or CSE/O-Level/GCSE or equivalent	29 593	528	1 ref	1 ref	
AS/A-Level or equivalent	11 008	201	1.01 (0.86–1.19)	0.99 (0.84–1.17)	
Higher education or other professional qualification, or equivalent	137 027	2797	1.07 (0.98–1.18)	1.04 (0.94–1.14)	
Ethnicity					<0.001
White	205 839	4374	1 ref	1 ref	
Mixed background	1077	13	0.95 (0.55–1.64)	1.01 (0.58–1.74)	
Asian	5765	49	0.57 (0.43–0.76)	0.62 (0.47–0.83)	
Black	3279	89	2.40 (1.94–2.97)	2.61 (2.10–3.24)	
Other	1926	23	0.94 (0.63–1.42)	1.02 (0.67–1.54)	
Unemployment					0.719
Paid/self-employment	134 112	1904	1 ref	1 ref	
Not in paid/self-employment	85 223	2671	1.00 (0.93–1.07)	1.01 (0.95–1.08)	
Lives with a wife or partner					0.331
No	52 368	945	1 ref	1 ref	
Yes	166 967	3630	1.08 (1.01–1.17)	1.04 (0.96–1.12)	
**Anthropometric**					
Height, cm					
<170	39 918	936	1 ref	1 ref	
⩾170–<175	55 888	1227	1.02 (0.94–1.11)	1.00 (0.92–1.09)	
⩾175–<180	60 939	1256	1.04 (0.96–1.13)	1.00 (0.92–1.09)	
⩾180–<185	40 076	786	1.10 (1.00–1.21)	1.05 (0.95–1.15)	
⩾185	21 175	355	1.10 (0.97–1.24)	1.04 (0.92–1.18)	
Per 10 cm increase	217 996	4560	1.06 (1.00–1.11)	1.03 (0.98–1.08)	0.307
BMI, kg m^−2^					
<25	53 707	1142	1 ref	1 ref	
⩾25–<30	107 927	2396	0.98 (0.92–1.06)	0.98 (0.92–1.06)	
⩾30–<35	43 500	834	0.85 (0.78–0.93)	0.88 (0.81–0.97)	
⩾35	12 796	188	0.69 (0.59–0.81)	0.75 (0.64–0.88)	
Per 5 kg m^−2^ increase	217 930	4560	0.89 (0.85–0.93)	0.91 (0.87–0.95)	<0.001
Body fat^d^, fifth cutoff %					
<20.5	43 803	816	1 ref	1 ref	
⩾20.5–<24	43 270	938	1.02 (0.93–1.12)	1.02 (0.93–1.12)	
⩾24–<26.8	42 957	949	0.95 (0.87–1.05)	0.96 (0.87–1.06)	
⩾26.8–<30.1	41 925	899	0.86 (0.78–0.95)	0.88 (0.80–0.97)	
⩾30.1	42 351	882	0.77 (0.70–0.84)	0.81 (0.73–0.89)	
Per 5% increase	214 257	4484	0.90 (0.88–0.93)	0.92 (0.90–0.95)	<0.001
Waist circumference,^d^ fifth cutoff cm					
<88	49 129	939	1 ref	1 ref	
⩾88.1–<93.1	39 113	826	0.99 (0.91–1.09)	1.00 (0.91–1.10)	
⩾93.1–<98.1	49 502	1113	1.00 (0.91–1.09)	1.01 (0.92–1.10)	
⩾98.1–<105.1	37 174	817	0.93 (0.85–1.02)	0.96 (0.87–1.05)	
⩾105.1	43 390	869	0.84 (0.76–0.92)	0.90 (0.82–0.99)	
Per 10 cm increase	218 308	4564	0.94 (0.91–0.97)	0.96 (0.93–0.99)	0.016
Waist to hip ratio,^d^ fifth cutoff					
<0.882	43 848	835	1 ref	1 ref	
⩾0.882–<0.918	43 787	835	0.87 (0.79–0.96)	0.88 (0.80–0.97)	
⩾0.918–<0.949	43 872	982	0.94 (0.86–1.03)	0.96 (0.88–1.06)	
⩾0.949–<0.989	43 110	964	0.87 (0.79–0.96)	0.91 (0.83–1.00)	
⩾0.989	43 639	948	0.80 (0.73–0.88)	0.87 (0.79–0.96)	
Per 0.05 increase	218 256	4564	0.94 (0.92–0.97)	0.97 (0.94–1.00)	0.021
**Lifestyle**					
Smoking					0.004
Never	107 188	2134	1 ref	1 ref	
Former	83 154	1991	0.90 (0.85–0.96)	0.93 (0.88–0.99)	
Current	27 428	417	0.82 (0.73–0.91)	0.85 (0.77–0.95)	
Alcohol intake, g per day					
None drinkers	13 874	225	0.83 (0.70–0.99)	0.85 (0.72–1.01)	
<1	14 577	299	1 ref	1 ref	
⩾1–<10	48 187	1030	1.08 (0.95–1.23)	1.04 (0.91–1.18)	
⩾10–<20	46 565	1071	1.11 (0.98–1.26)	1.06 (0.93–1.21)	
⩾20	94 750	1930	1.02 (0.90–1.15)	1.01 (0.89–1.14)	
Per 10 g per day increase	204 723	4340	0.98 (0.96–1.00)	0.99 (0.97–1.01)	0.405
Physical activity, METs per week					
Low, 0–<10	60 589	1196	1 ref	1 ref	
Moderate, ⩾10–<50	103 570	2244	1.10 (1.02–1.18)	1.06 (0.99–1.14)	
High, ⩾50	47 126	975	1.05 (0.96–1.14)	1.01 (0.93–1.10)	
Per 20 METs per week increase	211 070	4413	1.00 (0.98–1.03)	1.00 (0.98–1.02)	0.771

Abbreviations: AS=advanced subsidiary level; BMI=body mass index; CI=confidence interval; CSE=Certificate of Secondary Education; GCSE=General Certificate of Secondary Education; HR=hazard ratio; MET=metabolic equivalent of task; ref=reference.

Missing and/or unknown values of the exposure of interest were not included in the analyses, but missing and/or unknown values were assigned to a separate category when the variable was included as a covariate.

a Minimally adjusted model: HRs are stratified by region (10 UK cancer registry regions) and age at recruitment (<45, 45–49, 50–54, 55–59, 60–64, and ⩾65 years) and adjusted for age (underlying time variable), use as appropriate.

b Multivariable-adjusted model: HRs are stratified by region and age at recruitment and adjusted for age (underlying time variable), Townsend deprivation score (fifths, unknown), ethnicity (white, mixed background, Asian, black, other, and unknown), lives with a wife or partner (no and yes), BMI (<25, ⩾25–<30, ⩾30–<35, ⩾35 kg m^−2^, unknown), smoking (never, former, current, and unknown), physical activity (low (0–<10 METs per week), moderate (⩾10–<50 METs per week), high (⩾50 METs per week), and unknown), diabetes (no, yes, and unknown), enlarged prostate (no or unknown, and yes), and family history of prostate cancer (no, yes, unknown), use as appropriate.

c *P*-values from the multivariable-adjusted model were calculated as follows: *P*-values for likelihood ratio test for variables with more than two categories were obtained comparing the model with and without the variable of interest; *P*-values for dichotomous variables were obtained comparing the reference category to the other category in the model; *P*-values for trend were obtained using a pseudo-continuous variable equal to the median value in each category for continuous variables.

d Multivariable-adjusted model as above but without adjustment for BMI.

**Table 3:** Hazard ratios (95% CI) for prostate cancer in relation to various health status, prostate specific factors prior to recruitment, sexual history, and early life factors at baseline

**Characteristics**	**Total** ***n***	**Cases**	**HR (95% CI) minimally adjusted**^a^	**HR (95% CI) multivariable-adjusted**^b^	***P*****-value**^c^
**Health status**					
Vasectomy					0.662
No or unknown	207 969	4343	1 ref	1 ref	
Yes	11 366	232	1.05 (0.92–1.20)	1.03 (0.90–1.18)	
Hypertension					0.965
No or unknown	104 608	1866	1 ref	1 ref	
Yes	114 193	2700	0.99 (0.93–1.05)	1.00 (0.94–1.06)	
Diabetes					<0.001
No	202 785	4283	1 ref	1 ref	
Yes	15 191	270	0.65 (0.57–0.73)	0.70 (0.62–0.80)	
**Prostate-specific factors prior to recruitment**					
PSA test					<0.001
No	154 265	2283	1 ref	1 ref	
Yes	58 497	2009	1.43 (1.35–1.52)	1.31 (1.23–1.40)	
Enlarged prostate					<0.001
No or unknown	212 242	4216	1 ref	1 ref	
Yes	7093	359	1.57 (1.41–1.76)	1.54 (1.38–1.71)	
Any first-degree family history of prostate cancer					<0.001
No	103 961	1745	1 ref	1 ref	
Yes	16 449	599	1.96 (1.78–2.15)	1.94 (1.77–2.13)	
Family history of prostate cancer					
No	103 961	1745	1 ref	1 ref	<0.001
Father or brother	16 098	569	1.91 (1.74–2.10)	1.90 (1.72–2.09)	
Father and brother	351	30	3.51 (2.45–5.04)	3.35 (2.33–4.81)	
**Sexual history**					
Ever had children					0.007
Yes	171 957	3879	1 ref	1 ref	
Never	45 375	669	0.87 (0.80–0.94)	0.89 (0.81–0.97)	
Number of children					0.001
1	27 488	517	0.94 (0.85–1.03)	0.95 (0.86–1.04)	
2	90 098	2131	1 ref	1 ref	
⩾3	54 371	1231	0.92 (0.86–0.99)	0.93 (0.86–1.00)	
Ever had sexual intercourse					0.007
Yes	191 476	3999	1 ref	1 ref	
Never	1916	18	0.51 (0.32–0.81)	0.53 (0.33–0.84)	
Age at first sexual intercourse, years					0.024
<16	88 078	1781	0.93 (0.86–1.01)	0.97 (0.89–1.06)	
⩾16–<20	48 186	803	1 ref	1 ref	
⩾20–<25	42 717	1125	1.02 (0.94–1.09)	0.98 (0.91–1.06)	
⩾25	12 495	290	0.96 (0.84–1.08)	0.91 (0.80–1.03)	
Lifetime number of heterosexual partners					0.013
1	41 598	1126	1 ref	1 ref	
⩾2–<6	65 701	1392	0.96 (0.89–1.04)	0.99 (0.91–1.07)	
⩾6	68 971	1161	0.99 (0.91–1.07)	1.03 (0.95–1.13)	
Same-sex intercourse					0.171
No	189 406	3989	1 ref	1 ref	
Yes	8576	146	1.07 (0.90–1.26)	1.12 (0.95–1.33)	
Lifetime number of same-sex partners					0.964
1	977	18	1 ref	1 ref	
⩾2–<6	1340	25	1.05 (0.57–1.92)	1.05 (0.57–1.92)	
⩾6	1716	22	0.94 (0.50–1.75)	0.93 (0.50–1.74)	
**Early life**					
Relative age of first facial hair					0.454
Younger than average	14 262	243	1.01 (0.89–1.15)	1.03 (0.90–1.17)	
About average	167 220	3656	1 ref	1 ref	
Older than average	27 009	459	0.97 (0.88–1.06)	0.94 (0.86–1.04)	
Relative age voice broke					0.548
Younger than average	8769	143	0.99 (0.84–1.17)	1.00 (0.85–1.18)	
About average	178 309	3813	1 ref	1 ref	
Older than average	11 861	194	0.95 (0.83–1.10)	0.92 (0.80–1.07)	
Comparative body size at age 10					0.156
Thinner	75 069	1516	0.94 (0.88–1.00)	0.94 (0.88–1.00)	
About average	109 231	2414	1 ref	1 ref	
Plumper	29 305	536	0.92 (0.84–1.01)	0.96 (0.88–1.06)	
Comparative height size at age 10					0.986
Shorter	42 033	865	1.00 (0.93–1.08)	1.00 (0.92–1.08)	
About average	119 071	2510	1 ref	1 ref	
Taller	53 953	1115	1.01 (0.94–1.09)	1.00 (0.94–1.08)	
**Hair colour and pattern**					
Hair colour (natural, before greying in whites)					0.597
Light brown	79 825	1790	1 ref	1 ref	
Red	7726	144	0.88 (0.75–1.05)	0.88 (0.74–1.05)	
Blonde	19 935	418	0.95 (0.86–1.06)	0.96 (0.86–1.06)	
Dark brown	77 304	1523	0.98 (0.91–1.05)	0.97 (0.91–1.04)	
Black	16 718	406	1.02 (0.92–1.14)	1.03 (0.92–1.14)	
Other	17 152	287	1.01 (0.89–1.14)	0.99 (0.79–1.23)	
Hair/balding pattern^d^					0.595
Pattern 1	69 647	1317	1 ref	1 ref	
Pattern 2	49 303	903	0.99 (0.91–1.08)	0.99 (0.91–1.07)	
Pattern 3	57 208	1388	1.00 (0.93–1.08)	0.99 (0.92–1.07)	
Pattern 4	39 051	882	0.95 (0.87–1.03)	0.95 (0.87–1.03)	

Abbreviations: CI=confidence interval; HR, hazard ratio; PSA=prostate-specific antigen; ref, reference.

Missing and/or unknown values of the exposure of interest were not included in the analyses, but missing and/or unknown values were assigned to a separate category when the variable was included as a covariate.

a Minimally adjusted model: HRs are stratified by region (10 UK cancer registry regions) and age at recruitment (<45, 45–49, 50–54, 55–59, 60–64, and ⩾65 years) and adjusted for age (underlying time variable), use as appropriate.

b Multivariable-adjusted model: HRs are stratified by region and age at recruitment and adjusted for age (underlying time variable), Townsend deprivation score (fifths, unknown), ethnicity (white, mixed background, Asian, black, other, and unknown), lives with a wife or partner (no and yes), BMI (<25, ⩾25–<30, ⩾30–<35, ⩾35 kg m^−2^, and unknown), smoking (never, former, current, and unknown), physical activity (low (0–<10 METs per week), moderate (⩾10–<50 METs per week), high (⩾50 METs per week), and unknown), diabetes (no, yes, and unknown), enlarged prostate (no or unknown, and yes), and family history of prostate cancer (no, yes, and unknown), use as appropriate.

c *P*-values from the multivariable-adjusted model were calculated as follows: *P*-values for likelihood ratio test for variables with more than two categories were obtained comparing the model with and without the variable of interest; *P*-values for dichotomous variables were obtained comparing the reference category to the other category in the model; *P*-values for trend were obtained using a pseudo-continuous variable equal to the median value in each category for continuous variables.

d Pattern 1, no balding; pattern 2, balding at the front; pattern 3, balding on the top of head; pattern 4, complete balding.
