# Supplementary material for: Prospective investigation of risk factors for prostate cancer in the UK Biobank cohort study
Source: Br J Cancer. 2017 Sep 14;117(10):1562–71. doi: 10.1038/bjc.2017.312 (PMC5680461; doi:10.1038/bjc.2017.312)
Supplement: Supplementary Information [file bjc2017312x1.docx]

**SUPPLEMENTARY METHODS**

***Exposure assessment***

Sociodemographic characteristics

Participants provided detailed self-reported data via a touch screen questionnaire at the assessment centres at baseline. Socio-demographic factors included UK Biobank assessment centre (Barts, Hounslow, Croydon, Swansea, Wrexham, Cardiff, Stockport, Manchester, Liverpool, Bury, Newcastle upon Tyne, Middlesbrough, Leeds, Sheffield, Stoke, Birmingham, Nottingham, Oxford, Reading, Bristol, Glasgow, and Edinburgh), age at recruitment (month and year of birth as acquired by the central registry and updated by participant, with each participant nominally assigned 15 as the day of birth), education level (participants were asked “Which of the following qualifications do you have? (You can select more than one)” with possible answers being: “College or University degree”, “A levels/AS levels or equivalent”, “O levels/GCSEs or equivalent”, “CSEs or equivalent”, “NVQ or HND or HNC or equivalent”, “Other professional qualifications e.g. nursing, teaching”, “None of the above”) , ethnicity (participants were asked “What is your ethnic group?” with possible answers being “White”, “Mixed”, “Asian or Asian British”, “Black or “Black British”, “Chinese”, “Other ethnic group”, “Do not know”, “Prefer not to answer”, employment (participants were asked “Which of the following describes your current situation? (You can select more than one answer)” with possible answers being: “In paid employment or self-employed”, “Retired”, “Looking after home and/or family”, “Unable to work because of sickness or disability”, “unemployed”, “Doing unpaid or voluntary work”, “Full or part-time student”, “None of the above”, “Prefer not to answer”), and lives with a wife or partner (participants were asked “How are the other people who live with you related to you? (You can select more than one answer)” with possible answers being: “Husband, wife or partner”, “Son and/or daughter (include step-children)”, “Brother and/or sister”, “Mother and/or father”, “Grandparent”, “Grandchildren”, “Other related”, “Prefer not to answer”). Townsend deprivation index was calculated using participant’s home postal codes (Townsend P, 1988).

Anthropometric measurements

Anthropometric measurements were taken by trained research clinic staff (UK-Biobank, 2014). Standing height was measured to the nearest centimetre (cm), without shoes, using the SECA 240 Height Measure. Weight measurement was measured without shoes and outdoor clothing, using the Tanita BC 418 body composition analyser or using standard scales if the participant did not undergo bioimpedance analysis. Body mass index (BMI) was calculated as weight (kg)/height (m^2^). Percentage body fat was measured using the Tanita BC 418 body composition analyser. Waist and hip circumference were collected from participants in a horizontal plane using a Seca 200cm tape measure. We derived waist to hip ratio (WHR) by dividing waist circumference by hip circumference.

Lifestyle characteristics

Smoking status was self-reported, and based on current/past smoking status the participant was classified as never, former, or current smoker. The grams of alcohol consumption per day were calculated using the glasses of some types of alcoholic beverages (e.g. 20 g/pint of beer, 10 g/glass of red and white wine, and spirits) per week. The question “About how often do you drink alcohol?” was used to identify non-drinkers. Total physical activity was computed as the sum of walking (2.3 excess metabolic equivalents [METs]), moderate activity (3.0 excess METs) and vigorous activity (7.0 excess METs) (for at least 10 minutes continuously). We report excess METs, which represent the energy expenditure above that of an inactive person.

Health status

Most medical conditions and operations were identified in a verbal interview with a trained research clinic staff, in which vasectomy status was recorded. Hypertension was identified if the participant had a measured systolic blood pressure greater than 140 mmHg or diastolic pressure greater than 90 mmHg (mean over two readings; manual or automated). We used participants’ answers to the question “Has a doctor ever told you that you have diabetes?” to identify participants with diabetes.

Prostate specific factors prior recruitment

Men were considered to have had a prostate-specific antigen (PSA) test if they answered positively to the question “Have you ever had a blood test for prostate cancer (prostate specific antigen or PSA test)?”. Benign prostatic hypertrophy, and enlarged prostate were identified in a verbal interview with a trained research clinic staff. Participants were asked about family history of prostate cancer (father or brothers) using the touchscreen questionnaire which was completed at the Assessment Centre.

Sexual history

We used individuals’ answers to the questions “How many children have you fathered?”, “What was your age when you first had sexual intercourse?”, “About how many sexual partners have you had in your lifetime?”, “Have you ever had sexual intercourse with someone of the same sex?”, and “How many sexual partners of the same sex have you had in your lifetime?” to identify their sexual history.

Early life factors

Participants were asked on the touchscreen questionnaire “When did you start to grow facial hair?” and “When did your voice break?”, with possible answers being “about average”, “younger than average”, “older than average”, “Don’t know” and “Prefer not to answer”. Men also replied to the question “Which of the following best describes your hair/balding pattern?” with possible answers being “Pattern 1 (no balding)”, “Pattern 2 (balding at the front)”, “Pattern 3 (balding on the top of head)”, “Pattern 4 (complete balding)”, “Don’t know” and “Prefer not to answer”. Information on body size and height at age 10 was collected through the question “When you were 10 years old, compared to average would you describe yourself as:” with possible answers being “About average”, “Plumper”, “Thinner”, “Don’t know” and “Prefer not to answer” for body size, and “About average”, “Taller”, “Shorter”, “Don’t know” and “Prefer not to answer” for height. Men also replied to the question “What best describes your natural hair colour? (If your hair colour is grey, the colour before you went grey)” and the possible answers were “Blonde”, “Red”, “Light Brown”, “Dark Brown”, “Black”, “Don’t know” and “Prefer not to answer”.

***Statistical analysis***

Cox proportional hazards models were used to calculate Hazard ratios (HRs) and 95% confidence intervals (CIs) for prostate cancer risk according to region of recruitment (ten UK cancer registry regions [London, Wales, North-West England, North-Eastern England, Yorkshire and the Humber, West Midlands, East Midlands, South-East England, South-West England, Scotland], except for when region was the main exposure of interest), Townsend deprivation score (fifths, higher fifth corresponds to the most deprived area), education level (No qualifications or CSE/O-Level/GCSE or equivalent, AS/A-Level or equivalent, higher education or other professional qualification or equivalent), ethnicity (white [British, Irish, Any other white background], mixed background [white and black Caribbean/African, White and Asian, Any other mixed background], Asian [Asian or Asian British, Chinese, Indian, Pakistani, Bangladeshi, Any other Asian background], black [black or black British, Caribbean, African, Any other Black background], other [other ethnic group]), employment (not in paid/self-employment, paid/self-employment), and lives with a wife or partner (no, yes), Height (<170, ≥170-<175, ≥175-<180, ≥180-<185, and ≥185 cm), BMI (<25, ≥25-<30, ≥30-<35, ≥35 kg/m^2^), body fat (fifths), waist circumference (fifths), waist to hip ratio (fifths), smoking (never, former, current), alcohol intake (<1, ≥ 1- < 10, ≥ 10- < 20, ≥ 20 g/day), physical activity (low [0 - <10 METs/week], moderate [≥10 - <50 METs/week], and high [≥50 METs/week]), vasectomy (no or unknown, yes), hypertension (no or unknown, yes), diabetes (no, yes), PSA test (no, yes), enlarged prostate (no or unknown, yes [included those reporting having had benign prostatic hypertrophy]), any first degree family history of prostate cancer (no, yes [father or brother]), family history of prostate cancer (no, father or brother, father and brother) ever had children (yes, never), number of children (none, 1, 2, ≥ 3), ever had sexual intercourse (yes, never), age at first sexual intercourse (never had sex, < 16, ≥ 16 - < 20, ≥ 20 - < 25, ≥ 25 years), lifetime number of heterosexual partners (never had sex, 1, ≥ 2 - < 6, ≥ 6), same-sex intercourse (no, yes), lifetime number of homosexual partners (never had sex, 1, ≥ 2 - < 6, ≥ 6), relative age of first facial hair (about average, younger than average, older than average) relative age voice broke (about average, younger than average, older than average), hair/balding pattern (Pattern 1, Pattern 2, Pattern 3, Pattern 4), comparative body size at age 10 (about average, plumper, thinner), comparative height size at age 10 (about average, taller, shorter), hair colour (natural, before greying in whites) (light brown, red, blonde, dark brown, black, other). Age was used as the underlying time variable in all models.

**References**

Townsend P PP, Beattie A (1988) Health and Deprivation: Inequality and the North. London: Croom Helm.

UK-Biobank (2014) UK Biobank Anthropometry. Available at <http://biobank.ctsu.ox.ac.uk/crystal/docs/Anthropometry.pdf>.
