# Supplementary figures and images for: Prospective investigation of risk factors for prostate cancer in the UK Biobank cohort study
Source: Br J Cancer. 2017 Sep 14;117(10):1562–71. doi: 10.1038/bjc.2017.312 (PMC5680461; doi:10.1038/bjc.2017.312)

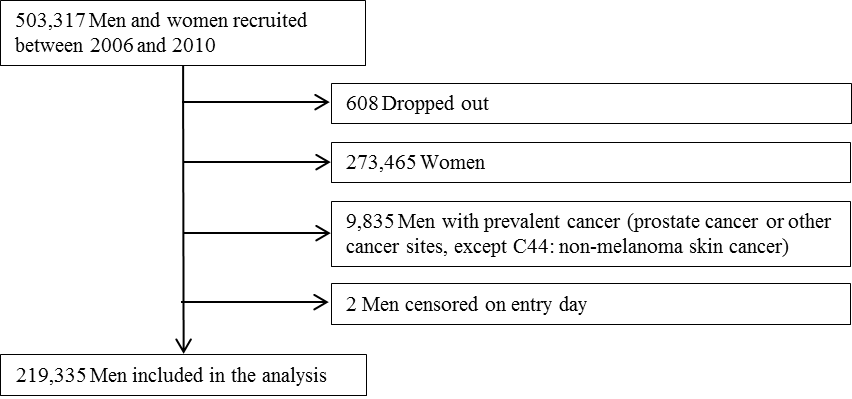


**Supplementary Figure 1**. Selection criteria of the study participants.

Supplement: Supplementary Figure 1 [file bjc2017312x2.docx]
