# Supplementary material for: Prospective investigation of risk factors for prostate cancer in the UK Biobank cohort study
Source: Br J Cancer. 2017 Sep 14;117(10):1562–71. doi: 10.1038/bjc.2017.312 (PMC5680461; doi:10.1038/bjc.2017.312)
Supplement: Supplementary Table 1 [file bjc2017312x3.docx]

| **Supplementary Table 1.**  Multivariable-adjusted hazard ratios (95% CI) for prostate cancer by time to diagnosis in relation to various sociodemographic, anthropometric, lifestyle, health status, prostate specific factors prior recruitment, sexual history and early life factors. | | | | | |
| --- | --- | --- | --- | --- | --- |
|  | **Time to diagnosis** | | | |  |
|  | **< 2 years** | | **≥ 2 years** | |  |
| **Characteristics** | **Cases**^1^  (exposed/unexposed) | **HR (95% CI)** | **Cases**  (exposed/unexposed) | **HR (95% CI)** | **P for het** |
| **Sociodemographic** |  |  |  |  |  |
| Townsend deprivation score |  |  |  |  |  |
| Above median  *vs* below median | 615 /815 | 0.85 (0.76 - 0.95) | 1,378 /1,762 | 0.94 (0.87 - 1.01) | 0.149 |
| Education |  |  |  |  |  |
| Higher education *vs* no qualifications or CSE/O-Level/GCSE or equivalent or AS/A-Level or equivalent | 879 /241 | 0.99 (0.86 - 1.14) | 1,918 /488 | 1.06 (0.96 - 1.17) | 0.427 |
| Ethnicity |  |  |  |  |  |
| Black *vs* White | 42 /1,361 | 3.94 (2.85 - 5.44) | 47 /3,013 | 2.01 (1.49 - 2.70) | 0.001 |
| Unemployment |  |  |  |  |  |
| Not in paid/self-employment *vs* paid/self-employment | 913 /519 | 1.34 (1.18 - 1.51) | 1,758 /1,385 | 0.89 (0.82 - 0.97) | <0.001 |
| Lives with a wife or partner |  |  |  |  |  |
| Yes *vs* No | 1,143 /289 | 1.08 (0.94 - 1.23) | 2,487 /656 | 1.02 (0.93 - 1.12) | 0.513 |
|  |  |  |  |  |  |
| **Anthropometry** |  |  |  |  |  |
| Height |  |  |  |  |  |
| Per 10 cm increase | 1,427 | 1.00 (0.92 - 1.09) | 3,133 | 1.05 (0.98 - 1.11) | 0.429 |
| BMI |  |  |  |  |  |
| Per 5 kg/m^2^ increase | 1,428 | 0.81 (0.69 - 0.95) | 3,132 | 0.84 (0.76 - 0.94) | 0.678 |
| Body fat |  |  |  |  |  |
| Per 5 % increase | 1,400 | 0.93 (0.88 - 0.98) | 3,084 | 0.92 (0.89 - 0.96) | 0.910 |
| Waist circumference |  |  |  |  |  |
| Per 10 cm increase | 1,429 | 0.97 (0.91 - 1.03) | 3,135 | 0.96 (0.92 - 1.00) | 0.738 |
| Waist to hip ratio |  |  |  |  |  |
| Per 0.05 increase | 1,429 | 1.00 (0.95 - 1.05) | 3,135 | 0.96 (0.92 - 0.99) | 0.168 |
|  |  |  |  |  |  |
| **Lifestyle characteristics** |  |  |  |  |  |
| Smoking |  |  |  |  |  |
| Current *vs* never or former | 124 /1,298 | 0.84 (0.69 - 1.01) | 293 /2,827 | 0.90 (0.80 - 1.02) | 0.496 |
| Alcohol intake |  |  |  |  |  |
| Per 10 g/day increase | 1,425 | 0.99 (0.95 - 1.03) | 3,130 | 0.99 (0.97 - 1.02) | 0.873 |
| Physical activity |  |  |  |  |  |
| Per 20 METs/week increase | 1,381 | 0.97 (0.93 - 1.01) | 3,034 | 1.01 (0.98 - 1.04) | 0.087 |
|  |  |  |  |  |  |
| **Health status** |  |  |  |  |  |
| Vasectomy |  |  |  |  |  |
| Yes *vs* no | 72 /1,360 | 1.01 (0.79 - 1.28) | 160 /2,983 | 1.04 (0.89 - 1.22) | 0.819 |
| Hypertension |  |  |  |  |  |
| Yes *vs* no | 877 /553 | 1.12 (1.00 - 1.24) | 1,823 /1,313 | 0.95 (0.89 - 1.02) | 0.018 |
| Diabetes |  |  |  |  |  |
| Yes *vs* no | 96 /1,331 | 0.79 (0.64 - 0.98) | 174 /2,952 | 0.66 (0.56 - 0.77) | 0.178 |
|  |  |  |  |  |  |
| **Prostate specific factors prior recruitment** |  |  |  |  |  |
| PSA test |  |  |  |  |  |
| *Yes vs no* | 739 /630 | 1.51 (1.35 - 1.70) | 1,394 /1,606 | 1.23 (1.14 - 1.33) | 0.003 |
| Enlarged prostate |  |  |  |  |  |
| Yes *vs* no | 152 /1,280 | 2.16 (1.82 - 2.56) | 207 /2,936 | 1.27 (1.10 - 1.46) | <0.001 |
| Family history of prostate cancer |  |  |  |  |  |
| Yes *vs* no | 201 /520 | 2.13 (1.81 - 2.50) | 398 /1,225 | 1.85 (1.66 - 2.08) | 0.175 |
|  |  |  |  |  |  |
| **Sexual history** |  |  |  |  |  |
| Number of children |  |  |  |  |  |
| None *vs* any | 207 /1,215 | 0.87 (0.74 - 1.01) | 462 /2,664 | 0.90 (0.81 - 1.00) | 0.713 |
| Ever had sexual intercourse |  |  |  |  |  |
| Never *vs* ever sex | 6 /1,244 | 0.57 (0.25 - 1.28) | 12 /2,755 | 0.51 (0.29 - 0.90) | 0.823 |
| Lifetime number of heterosexual partners |  |  |  |  |  |
| Above median *vs* below median | 652 /510 | 0.94 (0.83 - 1.06) | 1,433 /1,102 | 1.00 (0.92 - 1.09) | 0.414 |
| Same-sex intercourse |  |  |  |  |  |
| Yes *vs* no | 46 /1,243 | 1.09 (0.81 - 1.48) | 100 /2,746 | 1.14 (0.93 - 1.40) | 0.821 |
| Lifetime number of same-sex partners |  |  |  |  |  |
| Above median *vs* below median | 1,271 /11 | 1.15 (0.64 - 2.09) | 2,805 /33 | 0.83 (0.59 - 1.17) | 0.348 |
|  |  |  |  |  |  |
| **Early life factors** |  |  |  |  |  |
| Relative age of first facial hair |  |  |  |  |  |
| Older than average *vs* about average and younger | 137 /1,233 | 0.87 (0.73 - 1.04) | 322 /2,666 | 0.98 (0.87 - 1.10) | 0.281 |
| Relative age voice broke |  |  |  |  |  |
| Older than average *vs* about average and younger | 61 /1,254 | 0.90 (0.70 - 1.17) | 133 /2,702 | 0.94 (0.79 - 1.11) | 0.807 |
| Comparative body size at age 10 |  |  |  |  |  |
| Plumper *vs* about average and thinner | 180 /1,219 | 1.06 (0.90 - 1.24) | 356 /2,711 | 0.95 (0.85 - 1.07) | 0.281 |
| Comparative height size at age 10 |  |  |  |  |  |
| Taller *vs* about average and shorter | 364 /1,039 | 1.06 (0.94 - 1.19) | 751 /2,336 | 0.98 (0.91 - 1.07) | 0.338 |
|  |  |  |  |  |  |
| **Hair colour and pattern** |  |  |  |  |  |
| Hair colour (natural, before greying) |  |  |  |  |  |
| Red *vs* light brown | 36 /1,394 | 0.73 (0.52 - 1.02) | 108 /3,030 | 0.97 (0.80 - 1.17) | 0.149 |
| Hair/balding pattern^2^ |  |  |  |  |  |
| Patterns 3 and 4 *vs* Patterns 1 and 2 | 701 /707 | 0.96 (0.86 - 1.06) | 1,569 /1,513 | 0.99 (0.92 - 1.07) | 0.557 |
| PSA, Prostate-Specific Antigen. | | | | | |
| Multivariable-adjusted model: HR are stratified by region and age at recruitment and adjusted for age (underlying time variable), Townsend deprivation score (fifths, unknown), ethnicity (white, mixed background, Asian, black, other, unknown), lives with a wife or partner (no, yes), BMI (<25, ≥25-<30, ≥30-<35, ≥35 kg/m^2^, unknown), smoking (never, former, current, unknown), physical activity (low [0 - <10 METs/week], moderate [≥10 - <50 METs/week], and high [≥50 METs/week], unknown), diabetes (no, yes, unknown), enlarged prostate (no or unknown, yes), family history of prostate cancer (no, yes, unknown), use as appropriate. | | | | | |
| *P*-value from test for heterogeneity for the associations of the exposure variable with risk of prostate cancer categorized according to age at recruitment (<2 or ≥2 years). | | | | | |
| ^1^For dichotomous variables case numbers are for exposed/unexposed cases. | | | | | |
| ^2^Pattern 1, no balding; Pattern 2, balding at the front; Pattern 3, balding on the top of head; Pattern 4, complete balding. | | | | | |
